# Moderating Effect of Residential History on the Effects of a Fatherhood Program on Parenting Skills Satisfaction among Nonresident African American Fathers

**DOI:** 10.3390/healthcare6010013

**Published:** 2018-02-09

**Authors:** Yiqing Qian, E. Hill De Loney, Cleopatra Howard Caldwell

**Affiliations:** 1 Center for Research on Ethnicity, Culture, and Health, School of Public Health, University of Michigan, 1415 Washington Heights, 2858 SPH1, Ann Arbor, MI 48109-2029, USA; yqq@umich.edu; 2 Flint Odyssey House, Flint, MI 48502, USA; cpyd442@yahoo.com; 3 Department of Health Behavior and Health Education, School of Public Health, University of Michigan, Ann Arbor, MI 48109-2029, USA

**Keywords:** nonresident African American fathers, parenting skills satisfaction, residential history, the Fathers and Sons Program

## Abstract

Nonresident African American (AA) fathers sometimes face challenges to achieving satisfaction with their parenting skills, which may inhibit their motivations for parenting. Studies have found that residential history of fathers is associated with parental involvement; however, current fatherhood programs rarely consider the influence of different residential history on fathering. In the current study, we examined whether nonresident AA fathers’ residential history with their sons moderated their parenting skills satisfaction after participating in the Fathers and Sons Program. Our results indicated that after controlling for fathers’ pretest parenting skills satisfaction, age, education, marital status, employment, and ever lived with their son’s mother; there was a moderating effect of residential history on the intervention’s effects on posttest parenting skills satisfaction. The regression analyses showed that fathers in the intervention group who had lived with their son increased their parenting skills satisfaction more at posttest compared with fathers who had never lived with their sons. However, fathers in the comparison group who had lived with their sons had lower posttest parenting skills satisfaction. Future fatherhood programs for nonresident AA fathers should develop more nuanced group-specific interventions that consider residential history as a critical factor to enhance their parenting skills satisfaction as a strategy for improving father involvement.

## 1. Introduction

Parenting satisfaction, defined as parents’ perceptions of pleasure and gratification regarding the parenting role [1], has been studied in several important domains including satisfaction with a parent’s child-rearing skills and performance, satisfaction with a parent’s relationship with child, as well as satisfaction with the spouse performance as a parent [2,3]. Previous studies have found that paternal parenting satisfaction is associated with fathers’ motivations for engaging in multiple parenting tasks in infants’ and children’s development [4,5,6], as well as their parenting self-efficacy [5,6,7], “the degree to which the parent feels competent and confident in handling child problems” [8], which leads to competent parenting behaviors and health child development [9,10]. Specifically, among nonresident fathers, fathers’ satisfaction with his parenting skills, one domain of parental role identity [3], is reported to be positively associated with parenting involvement and coparenting interaction after separation with their children’s mothers [3,4,6,11]. For example, our previous study found that among nonresident African American fathers and sons, fathers’ satisfaction with their parenting skills based on several parenting tasks, including communication about risky behaviors, socialization about race, and communication about sex, benefited their pre-adolescent sons’ development when sons felt more satisfied with fathers’ engagement [4].

However, it is often challenging for many nonresident African American fathers to be involved in coparenting of their children and achieving high levels of parenting skills satisfaction as resident fathers since they most dependent on the relationship with the child’s mother to maintain a relationship with their child [11]. Although studies have found that compared to other racial groups in the United States, nonresident African American fathers show more parenting involvement [12,13], they often face multiple social and economic stress attributable to economic strains and racial discrimination, which make it difficult to pay child support and fulfill the traditional father role as provider [7,13,14]. These stressors contribute to many nonresident African American fathers lower levels of parental satisfaction, which is related to their motivation to be consistently involved in the parenting of their children who do not live with them.

One important factor for nonresident fathers’ involvement with parenting is whether they have ever lived with their children before becoming nonresidential [15,16]. If they have lived with their children, fathers may have had opportunities to develop relationships with their children that may be more meaningful when they no longer live together. Previous studies have found that nonresident fathers in the United States had more frequent contact with their children when the separation occurred when children were older [15,17], and prior marriage to their children’s biological mother as well as the fathers’ current marital status also positively influenced their parenting behaviors [15]. In addition, nonresident fathers with higher education and those with a good relationship with the biological mother had more frequent contact with their children than those lacking these characteristics [16,17]. Most of these studies, however, were conducted with white families. There is limited qualitative and quantitative studies that have been conducted among low-income and minority families.

When developing paternal parenting enhancement programs for nonresident African American fathers, it would be erroneous to view them as a monolithic group. However, many existing fatherhood programs designed for nonresident African American fathers have not considered the influence that different residential histories with their children may have on the fathers’ ability to engage in parenting and to gain satisfaction from the parenting experience [18,19]. Yet the experiences some fathers may have had with their children prior to becoming nonresidential may have implications for their ability to benefit or not benefit from participating in fatherhood programs designed to enhance their parenting skills and abilities. Because of the importance of satisfaction with their ability to parent for father involvement, understanding more about the conditions under which parenting skills satisfaction is more likely to be enhanced within an intervention program for nonresident African American fathers is critical for providing more nuanced support at multiple levels of influence.

In the current study, we aimed to study: (1) whether nonresident African American fathers’ satisfaction with their parenting skills could be enhanced through the Fathers and Sons Program and (2) if so, was this relationship moderated by the father’s residential history (i.e., whether fathers ever lived with their son). We hypothesized that parenting skills satisfaction among fathers could be enhanced and that fathers who had lived with their sons would gain more parenting skills satisfaction from participating in the Fathers and Sons intervention after controlling for fathers’ age, ever lived with son’s mother, marital status, education level, employment status, and parenting skills satisfaction at pretest than fathers who had never lived with their sons.

## 2. Materials and Methods

### 2.1. Study Design

This study used data from the Fathers and Sons Program. The Fathers and Sons Program is a community-based participatory research program developed and evaluated using a theory-based, culturally relevant, and family-centered prevention intervention approach. The specific purpose of the program was to prevent youth risky health behaviors through enhancing nonresident African American fathers’ parenting skills and strengthening their relationships with their 8- to 12-year-old biological sons. A quasi-experimental, nonequivalent group pretest-posttest design was used for the evaluation. The conceptual model of the Fathers and Sons Program incorporates the theory of reasoned action [20], social cognitive theory [21], models of social network and social support [22], race-related socialization [23], and racial identity [24]. The details of the conceptual models are described in previous publication [18]. We incorporated the social environment from social cognitive theory in our initial conceptual model, which suggests that fathers’ ability to monitor their sons would be captured as part of the social environment because negotiations with the mother of the child are critical aspects of their relationship [18,25]. Living arrangements was initially included as a background factor for predicting paternal monitoring. In the current study, we examined if some fathers may have benefited more from the Fathers and Sons Program because of different living arrangements that could have created different social environments for the fathers and sons from a family perspective. A total of 158 nonresident father-son dyads participated in the intervention, and 129 dyads participated in the comparison group.

### 2.2. Sample

The sample for the current study included only fathers who completed the pretest and posttest questionnaires for the intervention and comparison groups (*n* = 255). Table 1 presents the descriptive statistics for the sample and compares study characteristics for the intervention and comparison groups at pretest. At pretest, the average ages of fathers were 37 and 38 years in the comparison group and intervention groups, respectively. The two groups were not different in age, residential history with the son, marital status, and employment status, however, there were group differences in ever lived with their son’ mother and education level (see Table 1). We controlled for all these covariates in the multivariate analyses because they have been found to be related to our outcome variable of interest and the education and ever lived with mother variables differed between the two groups.

### 2.3. Procedure

Families were recruited from two Midwestern cities with similar characteristics. Data were collected from the fall of 2002 to the fall of 2006. We obtained consent for participation from the father and from the mother/guardian for the son. Assent was obtained from the son for his participation. The study was approved by the University of Michigan’s Health Sciences Institutional Review Board and the Institutional Review Board of Hurley Hospital in the city where the intervention was conducted (HUM00103935). We paid participants a nominal amount for their participation [4].

The intervention curriculum consists of 15 sessions. In addition to the first session (program introduction) and the final session (closing ceremony), there were 13-session activities designed to enhance knowledge, influence attitude, and practice skill-building in specific areas such as effective parent-child communication, parental monitoring, and preventing substance use. The details of the activities in each session are reported somewhere else [18]. Families in the comparison group did not receive an intervention program. The completed the pretest and the posttest questionnaires like the intervention group families. Pretest data were collected prior to substantive activities for Session 2, and posttest data were collected at the end of the intervention (Session 14).

### 2.4. Measures

Parenting skills satisfaction at pretest and at posttest. Fathers were asked two questions to assess their parenting skills satisfaction: (1) the satisfaction with their ability to supervise their sons and (2) their overall satisfaction with parenting skills in relation to their sons. The response scale ranged from 1 (very dissatisfied) to 4 (very satisfied). Since these two measures were significantly correlated (Pearson’s *r* = 0.64 at pretest and Pearson’s *r* = 0.66 at posttest), we created an index of parenting skills satisfaction by summing them up. This parenting satisfaction index ranged from 2 to 8.

Age at pretest. Father’s age was a continuous variable measured in years.

Ever lived with the son at pretest. Fathers were asked whether they had ever lived in the same household with their son. The responses included “0: No” and “1: Yes.”

Ever lived with the son’s mother at pretest. Fathers were asked whether they had ever lived in the same household with their son’s mother. The responses included “0: No” and “1: Yes.”

Marital status at pretest. Fathers’ current marital status was assessed with six categories including “married,” “unmarried but living with partner,” “widowed,” “divorced,” “separated,” and “never married”. We created a dichotomous variable for marital status by combining the “married” and “unmarried but living with partner” as “1: married/cohabitating” and merging other categories as “0: not married/not cohabitating.”

Education level at pretest. The choices for fathers’ educational level included “1: Junior high school or less;” “2: Some high school;” “3: Received high school diploma/GED;” “4: Some college/completed technical program;” “5: Received a college diploma;” “6: Some graduate school;” “7: Received a graduate degree.” Using high school education as the reference, we created a variable with three categories including “Less than high school” (“Junior high school or less” or “some high school”), “Received high school diploma/GED,” and “Higher than higher school” (“some college/completed technical program” to “Received a graduate degree”).

Employment status at pretest. Fathers were asked whether they were currently working, temporarily laid-off from a regular job, or not working. In this study, we merged the “temporarily laid-off” and “not working” groups into one category to form “unemployed.” The binary employment status thus includes two categories, “1: employed” and “0: unemployed.”

Intervention. A binary variable for intervention was included as “1: intervention group” and “0: comparison group.”

### 2.5. Statistical Methods

Analyses were conducted using SAS 9.4 (SAS Institute Inc., Cary, NC, USA). Means, standard deviation (SD), and percentages were used to present the descriptive statistics of sample characteristics key study variables. Differences in these variables between the comparison group and the intervention group were examined using *t*-test or Chi-squared test.

Two multivariate linear regression models were constructed to examine whether enhancing father’s satisfaction with his parenting skills at posttest through the Fathers and Sons Program was moderated by fathers’ residential history with his son while controlling other pretest covariates including age, ever lived with the son’s mother, marital status, education level, employment status, and parenting skills satisfaction. In Model 1, we tested the main effect of the intervention on father’s posttest parenting skills satisfaction while controlling for other pretest covariates. In Model 2, we added the interaction term of the intervention and ever lived with the son to test the moderating effect of father’s residential history with his son on the intervention’s effects on fathers’ posttest parenting skills satisfaction.

### 2.6. Missing Data

Six percentages or less of data were missing on each of the variables in this study. The variables with missing values included ever lived with the son (*n* = 5), ever lived with the son’s mom (*n* = 1), marital status (*n* = 3), education level (*n* = 3), and employment status (*n* = 2), parenting skills satisfaction at pretest (*n* =1), and parenting skills satisfaction at posttest (*n* = 18). We excluded those participants with missing values (*n* = 32) from this study, which is 11% of the number of participants in the Fathers and Sons Program. Based on *t*-test and Chi-squared analyses, we found that the fathers eliminated from the analyses were not different from those who remained in the study based on father’s age, ever lived with their son, ever lived with son’s mother, marital status, education level, employment status, and parenting skills satisfaction at pretest.

## 3. Results

At pretest, fathers’ parenting skills satisfaction was not different (*t* = −0.26, *p*-value = 0.79) between the intervention group (Mean = 6.4, SD = 1.4) and comparison groups (Mean = 6.3, SD = 1.6). At posttest, compared to the comparison group (Mean = 6.4, SD = 1.6), fathers in the intervention group (Mean = 6.9, SD = 1.0) had higher level of e parenting skills satisfaction (*t* = −2.86, *p*-value < 0.05). After the program, intervention group fathers reported 0.5-units increase in average, and the fathers in the comparison group only reported 0.1-units increase.

**Figure 1:**
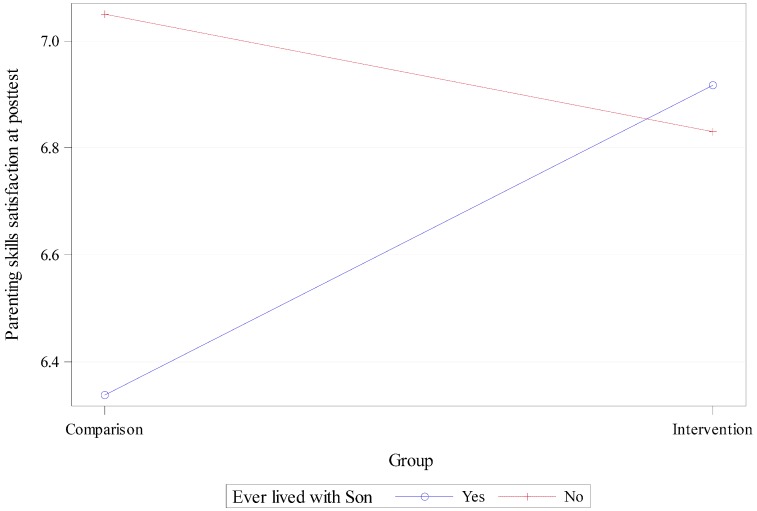
Residential history as a moderator of the Fathers and Sons intervention on parenting skills satisfaction at post-test among nonresident African American fathers. Note: Fit was computed at fathers aged 37.3 years old, ever lived mother, married/cohabitated, high school education level, employed, and pretest parenting satisfaction score of 6.3.

Table 2 presents the results of multivariate linear regression models. In Model 1 (*R*^2^ = 0.3733, *F* (9245) = 16.21, *p*-value < 0.0001), the intervention was positively associated with parenting satisfaction at posttest (β = 0.15, *p*-value < 0.005) when controlling for pretest covariates including age, ever lived with the son, ever lived with the son’s mother, marital status, education level, employment status, and parenting satisfaction. In Model 2 (*R*^2^ = 0.3892, *F* (10,244) = 15.55, *p*-value < 0.0001), this association was no longer significant (β = −0.08, *p*-value = 0.4364) when the interaction term between the intervention and ever lived with the son was added in Model 2. However, the interaction term was significant and positive (β = 0.30, *p*-value < 0.05). This indicates a moderating effect of fathers’ residential history with the sons on the effect of the Fathers and Sons intervention activities on enhancing the parenting skills satisfaction among the study participants. This moderating effect is further illustrated in Figure 1. As indicated, in the intervention group, fathers who had lived with their son showed higher parenting skills satisfaction at posttest compared with those who had never lived with their son. However, in the comparison group, the fathers who had lived with their sons had lower posttest parenting skills satisfaction than those fathers who had not lived with their sons.

## 4. Discussion

The results of our study show that the intervention activities in the Fathers and Sons Program enhanced the parenting skills satisfaction among nonresident African American fathers of preadolescent sons. We also found, however, that the effect of the intervention program was moderated by residential history with sons. In the intervention group, fathers who had lived with their son increased their parenting skills satisfaction more after the intervention compared with fathers who had never lived with their sons. Fathers in the comparison group who had lived with their sons, however, had lower posttest parenting skills satisfaction than those who had not lived with their sons.

Satisfaction with one’s parenting skills is one domain of the parental role identity [3], which is associated with more parenting involvement [26]. Higher parenting skills satisfaction is linked to more parenting involvement and better father-child relationship [3,4,6,11], which have significant influence on the development and well-being of children [4,18,27,28] and fathers [29,30]. Studies have found that nonresident fathers’ involvement was associated with less externalizing behaviors [4,18,27,28] as well as better academic performance [27] among children. Moreover, our previous study found that higher parenting skills satisfaction and better father-son relationship were positively associated with lower depressive symptoms among nonresident African American fathers [30]. The findings in this study confirm that parenting skills satisfaction is an appropriate focus for intervention programs with nonresident African American fathers [9].

The moderating effect of residential history on the program’s ability to improve parenting skills satisfaction among nonresident fathers is consistent with other studies of nonresident fatherhood [15,16,17]. For example, fathers who became nonresidential when children were younger were less involved with their children after the father-child separation [15,16]. Studies also reported that it was difficult for fathers to have close relationship with their nonresident adult children if the children did not have memory of living the fathers in early childhood [15]. Perhaps for nonresident African American fathers who had lived with their sons, they had an opportunity to form emotional bonds that could be the foundation for building more enduring relationships when both fathers and sons participated in the intervention program as a family. During the Fathers and Sons Program, it may have been easier for these fathers to continue bonding with their sons and generated a sense of parenting satisfaction and competence rarely experienced by nonresident fathers who are often expected to fail as parents by multiple sources of social and structural influences (e.g., mothers, social agencies, friends and family members, and even themselves). This study suggests that residential history with sons should be given more attention in the program design for paternal parenting enhancement programs among nonresident fathers.

More importantly, this study suggests that further investigation is needed on what types of intervention and specific contents that would be most beneficial for enhancing parenting skills satisfaction among nonresident African American fathers who have never lived with their sons or have only very limited residential history with the sons. This sub-group of nonresident fathers may need more foundational relationship building information and additional resources to achieve a level of parenting satisfaction to motivate sustained father involvement, especially when children are younger. Policy makers and service providers should not consider nonresident African American fathers a monolithic group as their needs are different across a multitude of lived experiences that should be taken into considerations in efforts to improve their lives and the lives of their children and families.

The current study has several limitations. First, we may not have a representative sample of nonresident African American fathers, due to possible selection biases from the convenience sampling strategy used for the Fathers and Sons Program. For example, fathers who enrolled in the study may be more engaged fathers than those who were reached out but chose not to enroll. Second, we were not able to know the long-term effect of the intervention since no follow-up surveys were designed after the Fathers and Sons Program ended. We are currently conducting a new and larger Fathers and Sons Study in Chicago with randomization of group assignment and longitudinal study design, i.e., several follow-ups after the program ends. We hope that the data collected from the new study will help us better understand the relationship between residential history and the long-term effects of the intervention among nonresident African American fathers and sons. Another limitation of this analysis is that the model estimates were subject to several untested confounders, such as the income of the fathers, which was not included in the Fathers and Sons Program questionnaires due to the sensitive nature of this question for minority population who may suffer from financial stress [13,14]. We also did not know the son’s age when the father-son separation occurred. The relationship between the nonresident father and the sons’ mothers is another potential confounder that is important in paternal parenting involvement outside marriage [11]. In the Fathers and Sons Program, although the mothers provided consent for their sons to participate, we did not obtain data from them that may have been helpful in avoiding method variance with some variables. Nevertheless, the findings of this study provide an opportunity to determine the conditions under which a fatherhood intervention program specifically designed for nonresident African American fathers can enhance their parenting skills satisfaction.

## 5. Conclusions

It is critical to encourage nonresident African American fathers to be involved in parenting [31]. Good father-son relationships benefit both nonresident fathers and their sons [4,18,27,28,29,30,31]. We suggest that future intervention programs for nonresident African American fathers could include more group-specific interventions and resources to increase their parenting skills satisfaction as a strategy to increase father involvement.

## Figures and Tables

**Table 1:** Descriptive statistics of study variables in the comparison and intervention groups.

Variable	Comparison (*n* = 107)		Intervention (*n* = 148)		*T*-test/Chi-Square Test (*p*-value)
	Mean (SD)	Frequency (%)	Mean (SD)	Frequency (%)	
Age	37.1 (8.0)		37.5 (7.3)		−0.39 (0.70)
Parenting skills satisfaction at pretest	6.3 (1.6)		6.4 (1.4)		−0.26 (0.79)
Ever lived with son					2.94 (0.09)
No		21 (19.6)		43 (29.1)	
Yes		86 (80.4)		105 (70.9)	
Ever lived with son’s mother *					6.51 (0.01)
No		13 (12.1)		37 (25.0)	
Yes		94 (87.9)		111 (75.0)	
Marital status					3.53 (0.06)
Not married/not cohabitating		67 (62.3)		109 (73.7)	
Married/cohabitating		40 (37.4)		39 (26.4)	
Education level *					9.06 (0.01)
Less than high school		22 (20.6)		34 (23.0)	
Received high school diploma/GED		22 (20.6)		53 (35.8)	
Higher than high school		63 (58.9)		61 (41.2)	
Employed					0.08 (0.77)
No		54 (50.5)		72 (48.6)	
Yes		53 (49.5)		76 (51.4)	

Note: * *p*-value < 0.05; ** *p*-value < 0.01.

**Table 2:** Multivariate regression analyses on parenting skills satisfaction at posttest.

Variable	Model 1	Model 2
	β	β
Intervention		
Yes	0.15 **	−0.08
No	0	0
Parenting skills satisfaction at pretest	0.56 ***	0.55 ***
Ever lived with the son		
Yes	−0.08	−0.24 *
No	0	0
Age	−0.03	−0.04
Ever lived with the son’s mom		
Yes	−0.05	−0.07
No	0	0
Marital status		
Married/Cohabitated	−0.03	−0.04
Not married/cohabitated	0	0
Education level		
Received high school diploma/GED	−0.04	−0.05
Higher than high school	−0.07	−0.08
Less than high school	0	0
Employment status		
Employed	0.09	0.09
Unemployed	0	0
Intervention * Ever lived with the son		0.30 *
*R*-squared	0.3733	0.3892
*R*-squared change		0.0159 *

Note: β, standardized coefficient; * *p*-value < 0.05; ** *p*-value < 0.01; *** *p*-value < 0.001.
